# Study protocol of HGCSG1404 SNOW study: a phase I/II trial of combined chemotherapy of S-1, nab-paclitaxel and oxaliplatin administered biweekly to patients with advanced gastric cancer

**DOI:** 10.1186/s12885-017-3850-z

**Published:** 2017-12-08

**Authors:** Yasuyuki Kawamoto, Yoshito Komatsu, Satoshi Yuki, Kentaro Sawada, Tetsuhito Muranaka, Kazuaki Harada, Hiroshi Nakatsumi, Hiraku Fukushima, Atsushi Ishiguro, Masayoshi Dazai, Kazuteru Hatanaka, Michio Nakamura, Ichiro Iwanaga, Minoru Uebayashi, Susumu Sogabe, Yoshimitsu Kobayashi, Takuto Miyagishima, Kota Ono, Naoya Sakamoto, Yuh Sakata

**Affiliations:** 1 Department of Cancer Center, Hokkaido University Hospital, Sapporo, Japan; 2 Department of Gastroenterology and Hepatology, Hokkaido University Graduate School of Medicine, Sapporo, Japan; 3 Department of Gastroenterology, JCHO, Sapporo Hokushin Hospital, Sapporo, Japan; 4 Department of Medical Oncology, Teine Keijinkai Hospital, Sapporo, Japan; 5 Department of Gastroenterology, Sapporo Medical Center NTT EC, Sapporo, Japan; 6 Department of Gastroenterology, Hakodate Municipal Hospital, Hakodate, Japan; 7 Department of Gastroenterology, Sapporo City General Hospital, Sapporo, Japan; 8 Department of Gastroenterology, Japanese Red Cross Kitami Hospital, Kitami, Japan; 9 Department of Internal Medicine, Kushiro Rosai Hospital, Kushiro, Japan; 10 Hokkaido University Hospital Clinical Research and Medical Innovation Center, Sapporo, Japan; 11 CEO, Misawa City Hospital, Misawa, Japan

**Keywords:** Gastric cancer, Chemotherapy, S-1, Nab-paclitaxel, Oxaliplatin

## Abstract

**Background:**

In Japan, S-1 plus cisplatin (SP) regimen has become a standard therapy for patients with advanced gastric cancer. Moreover, the S-1 plus oxaliplatin regimen is now a standard treatment.

Nab-paclitaxel was developed for chemotherapy of gastric cancer in Japanese clinical practice. Nab-paclitaxel, created with albumin-bound paclitaxel particles, has high transferability to tumour tissues and does not cause hypersensitivity reactions because of a different chemical composition compared with docetaxel and paclitaxel.

A combination of S-1, nab-paclitaxel and oxaliplatin (which we named ‘SNOW regimen’) can be a promising triplet therapy for advanced gastric cancer. Although we have to pay attention to chemotherapy-induced neuropathy, we aim to investigate the recommended dose of this regimen in a phase I study. Furthermore, we will investigate its efficacy and toxicity in a phase II study.

**Methods:**

The phase I study is a dose-escalation study using a standard 3 plus 3 design, followed by expansion cohorts. The SNOW regimen involves 28-day cycles with escalated doses of nab-paclitaxel (100–175 mg/m^2^ on days 1 and 15) and fixed doses of oxaliplatin (65 mg/ m^2^ on days 1 and 15) and S-1 (80 mg/m^2^/day on day 1 to 14). The primary endpoints are assessment of dose limiting toxicities and determination of maximum tolerated dose to investigate the recommended dose in the subsequent phase II study. In the phase II study, the primary endpoint is objective response rate. Secondary endpoints are assessment of safety, progression-free survival, disease control rate, overall survival and time to treatment failure. Adverse events were monitored and graded according to the National Cancer Institute Common Terminology Criteria for Adverse Events version 4.0.

**Discussion:**

Triplet therapies for advanced gastric cancer patients have been evaluated in clinical trials. The SNOW regimen can be a promising new triplet therapy.

**Trial registration:**

This study is performed at institutes that participate in Hokkaido Gastrointestinal Cancer Study Group (HGCSG) and registered as UMIN000016788. Registrated 16 March 2015.

## Background

In 2012, gastric cancer was the third leading cause of cancer deaths worldwide, responsible for 723,000 deaths [1]. Approximately 60% of gastric cancer patients worldwide are diagnosed in East Asian countries (Japan, China and Korea) [2].

Standard cytotoxic chemotherapy is frequently used as a first-line treatment for advanced gastric cancer (AGC), with a median overall survival of 8–12 months. The survival benefit with chemotherapy is not yet adequate; therefore, new agents and combination therapies are needed to improve the outcome of patients with advanced disease.

In patients with human epidermal growth factor receptor type 2 (HER2)-positive AGC, trastuzumab demonstrated a survival benefit in the ToGA study [3]. Trastuzumab in combination with cisplatin plus capecitabine or fluorouracil is a worldwide standard treatment for HER2-positive AGC. Meanwhile, for AGC without HER2 overexpression, several doublet or triplet first-line chemotherapy regimens, including fluorouracil, platinum, anthracycline or taxanes, are available [4, 5]. However, especially in triplet regimens, toxicity profiles must be carefully considered.

S-1 is an oral anticancer drug that combines tegafur, a prodrug of fluorouracil, with 5-chloro-2,4-dihydropyrimidine (CDHP) and oteracil potassium in a molar ratio of 1:0.4:1. CDHP reversibly antagonizes the activity of dihydropyrimidine dehydrogenase, the rate-limiting enzyme for the degradation of fluorouracil [6].

In Japan, the S-1 plus cisplatin (SP) regimen has become a standard therapy for patients with AGC [7]. Moreover, the S-1 combined with oxaliplatin (SOX) regimen has become a standard treatment [8].

Nab-paclitaxel was also developed for chemotherapy of gastric cancer in Japanese clinical practice [9]. Nab-paclitaxel, which is created with albumin-bound paclitaxel particles, has high transferability to tumour tissues and hardly cause a hypersensitivity reaction because of different chemical composition compared with docetaxel and paclitaxel.

To further improve the antitumor efficacy, the DCS regimen (SP combined with docetaxel) is considered as one of promising candidate of new standard treatment. And we have devised an S-1, nab-paclitaxel and oxaliplatin combination (which we named the ‘SNOW regimen’) which we have changed docetaxel to nab-paclitaxel, and cisplatin to oxaliplatin. That could be a promising triplet therapy for AGC patients. Although we have to pay attention to chemotherapy-induced neuropathy, we are aiming to investigate the recommended dose of this regimen in a phase I study. Furthermore, we will consider the efficacy and toxicity of this regimen in a phase II study.

## Methods/Design

### Inclusion criteria

1) Unresectable advanced, metastatic, or recurrent gastric or gastroesophageal junction cancer that is pathologically diagnosed as adenocarcinoma.

2) HER2 negative [0, 1+ in immunohistochemistry (IHC) or 2+ in IHC and FISH negative].

3) Patients who have measurable lesions based on Response Evaluation Criteria in Solid Tumors (RECIST) version 1.1.

4) Patients who have received no prior chemotherapy or radiotherapy for gastric or gastroesophageal junction cancer (patients who have received adjuvant therapy including S-1 before 180 days or more can be eligible; however, patients who have received adjuvant therapy including oxaliplatin at any time cannot be eligible).

5) 20 years of age and older.

6) Patients with Eastern Cooperative Oncology Group Performance Status of 0 or 1.

7) Patients who have possibility of oral intake.

8) Patients must have sufficient organ function as below:

- Absolute neutrophil count ≥1500/mm^3^
- Platelet count ≥100,000/mm^3^
- Haemoglobin ≥9.0 g/dL
- Total bilirubin ≤1.5 mg/dL
- Aspartate aminotransferase ≤100 U/L (≤ 200 in patients with liver metastases)
- Alanine aminotransferase ≤100 U/L (≤ 200 in patients with liver metastases)
- Serum creatinine ≤1.2 mg/dL
- Creatinine clearance ≥60 mL/min

9) Patients with a life expectancy of at least three months.

10) Patients must provide written informed consent.

### Exclusion criteria

1. Patients with history of hypersensitivity to any drugs in this study.
2. Patients with active infection.
3. Patients who are hepatitis B antigen positive.
4. Patients with serious complications, such as

- Uncontrollable cardiovascular disease, angina and arrhythmia
- Myocardial infarction in past three months
- Uncontrollable diabetes mellitus
- Intestinal lung disease or pulmonary fibrosis

5) Patients with ≥Grade 2 peripheral neuropathy.
6) Patients with any central nervous system metastases that are symptomatic or required treatment.
7) Patients with uncontrollable diarrhoea.
8) Patients with multiple primary cancers.
9) Female patients who are pregnant or lactating, or planning to become pregnant or lactating.
10) Other patients who are considered to be unsuitable for this study by the investigator.

### Treatment

**Fig. 1:**
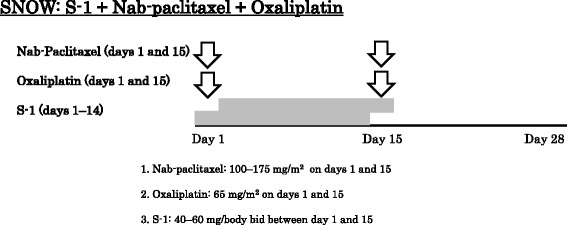
SNOW regimen. SNOW regimen comprises 28-day cycles with nab-paclitaxel and oxaliplatin on days 1 and 15, and S-1 (80 mg/m2/day on days 1–14)

The SNOW regimen consists of 28-day cycles with escalated doses of nab-paclitaxel (100–175 mg/m^2^ on days 1 and 15) and fixed doses of oxaliplatin (65 mg/m^2^ on days 1 and 15) and S-1 (80 mg/m^2^/day on day 1 to 14) **(**Fig. 1 **)**. In the setting of the administration schedule of the triplet regimen, the point where given in divided doses might relieve chemotherapy-induced neuropathy than both drugs gave a high dose in once, because nab-paclitaxel had an adverse event of chemotherapy-induced neuropathy and oxaliplatin coming at the same time was considered. The administration every two weeks of oxaliplatin was frequently used for FOLFOX therapies for colorectal cancer. And there was a report used by the administration method with 175 mg/m^2^ and 220 mg/m^2^ as preoperative chemotherapy as multidrug therapy for breast cancer for every two weeks about nab-paclitaxel. For these reason, we decided to set a dose in this schedule. For gastric cancer, oxaliplatin is approved in 130 mg/m^2^ every 3 weeks, and the dose concerned is 43.3 mg/m^2^ a week. Because this study was combination therapy, and chemotherapy-induced neuropathy might strongly develop by combination with nab-paclitaxel, we set 65 mg/m^2^ every 2 weeks at a fixation dose with a dose of oxaliplatin.

### Phase I part

**Fig. 2:**
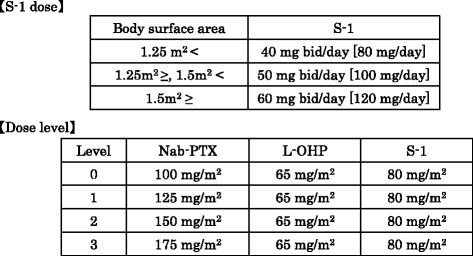
Dose-escalation in phase I part of SNOW regimen. In part of phase I study, escalated dose of nab-paclitaxel (100–175 mg/m2 on days 1 and 15), fixed dose of oxaliplatin (65 mg/ m2 on days 1 and 15) and S-1 (80 mg/m2/day on days 1–14) are administered

**Fig. 3:**
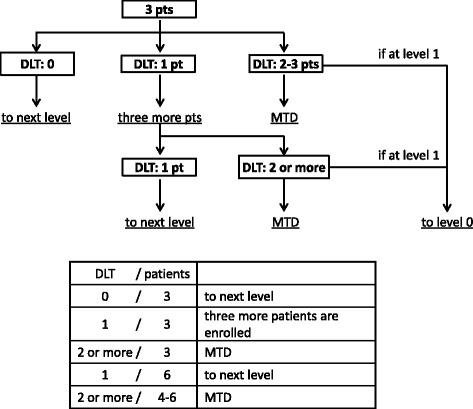
Flow chart in phase I part of SNOW regimen. In part of phase I study, this is a dose-escalation study using a standard 3 plus 3 design followed by expansion cohorts

The phase I part of the study is a dose-escalation study using a standard 3 plus 3 design followed by expansion cohorts as below **(**Figs. 2 and 3 **)**.

- We start at level 1.
- The recommended dose (RD) is defined as one dose level lower than the maximum tolerated dose (MTD).
- If 1 of three patients experienced dose-limiting toxicities (DLT), three more patients were enrolled at the same dose level.
- The MTD is defined as the dose level at which two or more of three patients, or at least two of 4–6 patients, had DLTs during cycle 1.

Adverse events are monitored and graded according to NCI-CTCAE (the National Cancer Institute Common Terminology Criteria for Adverse Events) version 4.0. The following adverse drug reactions are defined as DLT:

- ≥Grade 3 febrile neutropenia
- Grade 4 thrombocytopenia
- Grade 4 neutropenia over 7 days
- ≥Grade 3 non-haematological toxicities (excludes nausea and vomiting)
- delay of starting cycle 2 longer than 15 days due to adverse event

The primary endpoints are assessment of DLTs and determination of MTD to investigate the RD in subsequent phase II study.

**Fig. 4:**
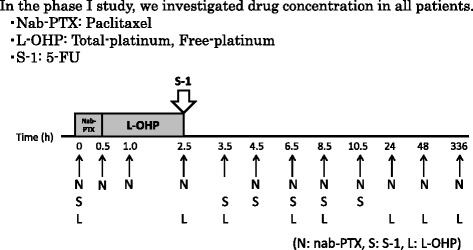
Pharmacodynamics study in phase I part of SNOW regimen. In the phase I study, we also investigated drug concentration in all patients. We measured paclitaxel concentration from nab-paclitaxel, total-platinum and free-platinum from L-OHP and 5-FU from S-1

In the phase I study, we also investigate drug concentrations in all patients (Fig. 4 **)**.

### Phase II part

In the phase II study, the primary endpoint is objective response rate according to RECIST version 1.1. We define the ratio of patients who are complete response or partial response in the overall study treatment time. Secondary endpoints are the assessment of adverse events graded according to NCI-CTCAE version 4.0, disease control rate, progression-free survival (PFS), time to treatment failure (TTF) and overall survival (OS). We evaluate PFS, TTF and OS using the Kaplan–Meier method.

### Estimated number of enrollments

In phase I, 3 to 6 patients are enrolled at each dose level (maximum of 18 patients). In phase II, we referred to the SOX [8] and DCS [10] regimens. The response rate was 58.8% (95% CI; 44.2–72.4%) for SOX and 81.4% (95% CI; 69.1–90.3%) for DSC. With a threshold response rate of 59% and an expected response rate of 81%, the simulation results indicated a sample size of 45 with α = 0.05 (both sides) for a power of 90% based on One Arm Binomial of SWOG. With an estimated dropout of some cases, a target sample size of 50 was estimated. That includes the cases administered the RD in phase I.

## Discussion

Triplet therapies for AGC patients have been evaluated in clinical trials [4, 5, 10–12]. The SNOW regimen comprising an S-1, nab-paclitaxel and oxaliplatin combination could be a promising triplet therapy.

Oxaliplatin often causes neuronopathy-type peripheral sensory neuropathy. Meanwhile, nab-paclitaxel causes axonopathy-type peripheral sensory neuropathy. The combination of oxaliplatin and nab-paclitaxel might cause severe chemotherapy-induced peripheral neuropathy; therefore, we have to be vigilant for signs of neuropathy. Administration of nab-paclitaxel in divided doses might reduce the neuro-toxicity.

This study is performed at institutes that participate in the Hokkaido Gastrointestinal Cancer Study Group (HGCSG) and is registered as UMIN000016788. Registrated 16 March 2015.

## Abbreviations

AGC: Advanced gastric cancer
CDHP: 5-chloro-2,4-dihydropyrimidine
DLT: Dose-limiting toxicities
HER2: Human epidermal growth factor receptor type 2
IHC: Immunohistochemistry
MTD: Maximum tolerated dose
OS: Overall survival
PFS: Progression-free survival
RD: Recommended dose
TTF: Time to treatment failure
